# Supplementary material for: Morphological and Molecular Identification of Ulva spp. (Ulvophyceae; Chlorophyta) from Algarrobo Bay, Chile: Understanding the Composition of Green Tides
Source: Plants (Basel). 2024 Apr 30;13(9):1258. doi: 10.3390/plants13091258 (PMC11085182; doi:10.3390/plants13091258)
Supplement: Supplementary file 1 [file plants-13-01258-s001.zip › Figure S1.pdf]

Figure S1. Voucher specimens of *Ulva* spp. collected from Los Tubos beach, Algarrobo Bay, Chile, housed at the National Museum of the Natural History, Chile SGO 171635 – 171651.

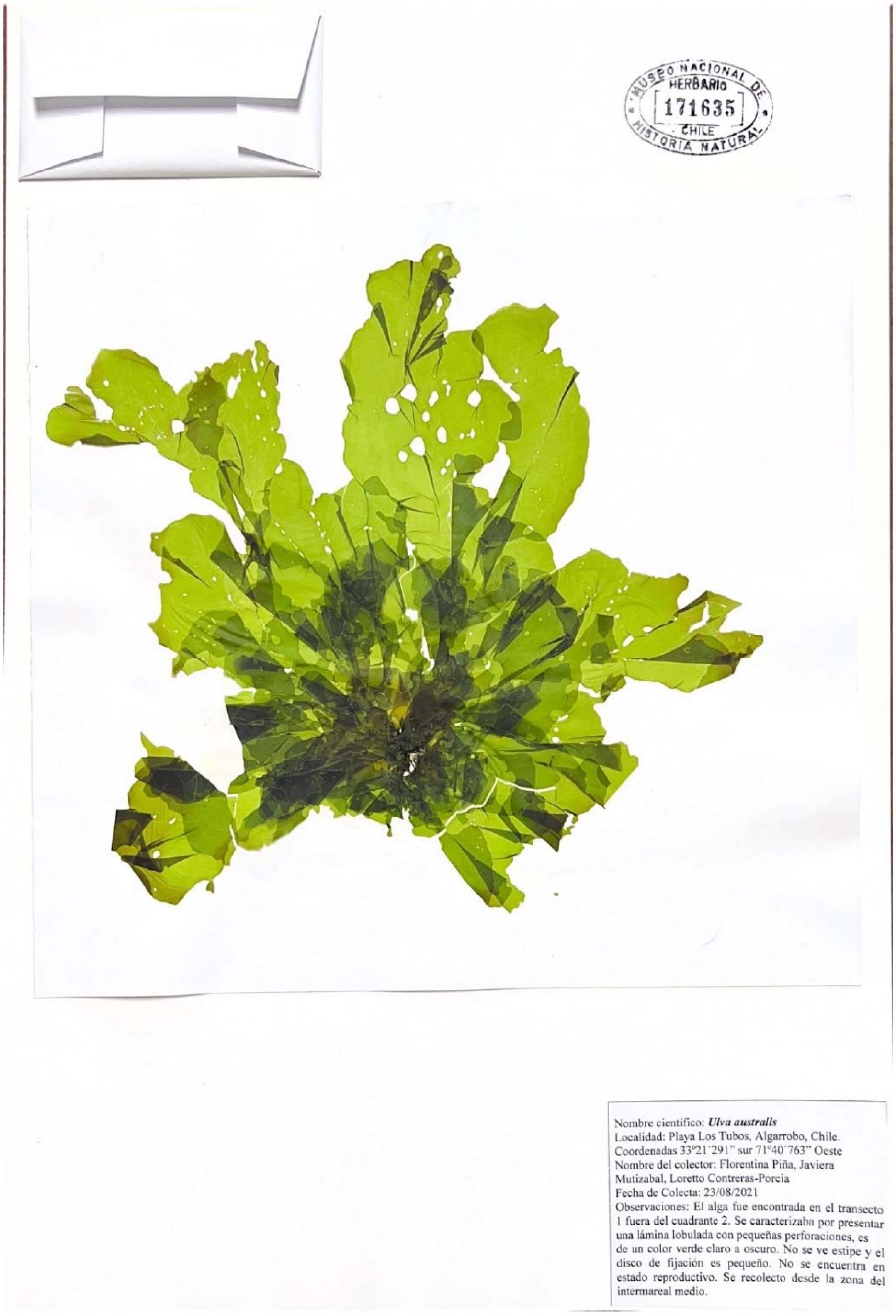

MUSEO NACIONAL DE HISTORIA NATURAL
HERBARIO
171649
CHILE

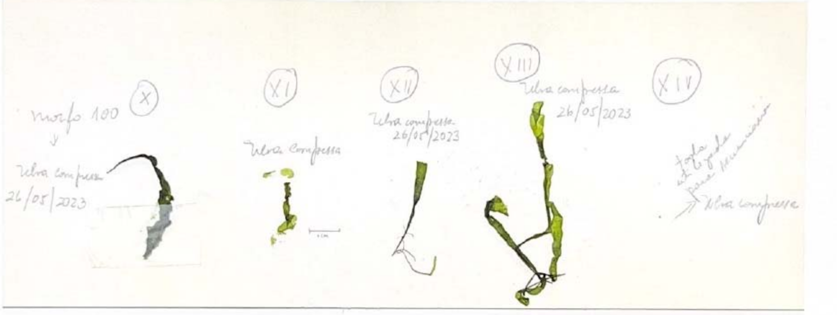

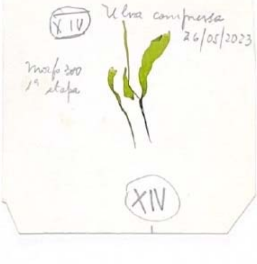

Nombre científico: *Ulva compressa*
Localidad: Playa Los Tubos, Algarrobo, V Región, Chile.
Coordenadas 33°21'891" sur 71°40'763" Oeste
Nombre del colector: Javiera Mutizabal, Alejandra Núñez y Loretto Contreras-Porcia
Fecha de Colecta: 11/03/2022
Observaciones: El alga fue encontrada en el sector su de la playa de Los Tubos (X-XIV); se caracterizan por presentar un talo tubular de color verde claro, delgado y suave al tacto, que se va engrosando hacia la parte superior. Se adhiere al sustrato mediante un disco muy pequeño de forma redonda
En su fronda se acumulan pequeños granos de arena.
Hábitat: se encuentra principalmente en el intermareal medio-alto en pozas con fondo arenoso.
Estado reproductivo: no se observa estructuras, por lo que se encuentra en una etapa vegetativa.

MUSEO NACIONAL DE HISTORIA NATURAL
HERBARIO
171651
CHILE

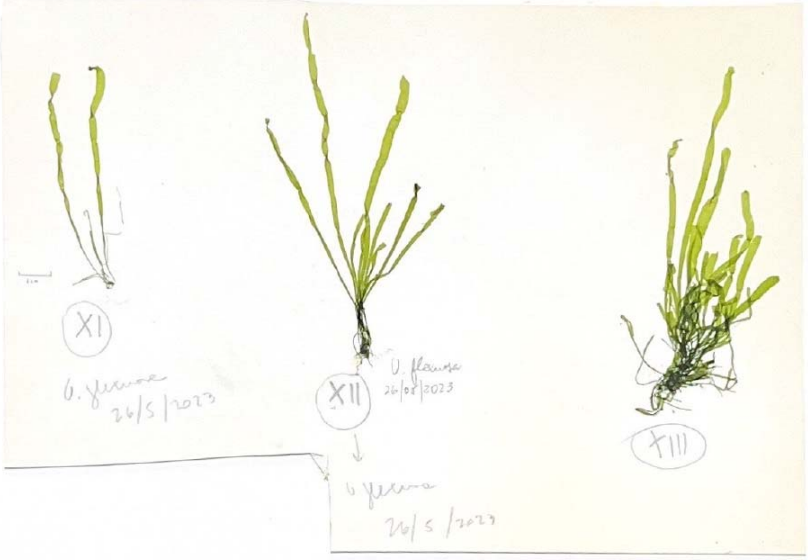

Nombre científico: ***Ulva flexuosa***
Localidad: Playa Los Tubos, Algarrobo, V Región, Chile.
Coordenadas 33°21`891" sur 71°40`763" Oeste
Nombre del colector: Javiera Mutizabal, Alejandra Núñez y Loretto Contreras-Porcia
Fecha de Colecta: 11/03/2022
Observaciones: El alga fue encontrada en el sector su de la playa de Los Tubos (X-XIV); se caracterizan por presentar un talo tubular de color verde claro, delgado, manteniendo su grosor desde el disco hasta la zona apical y en algunos casos ligeramente más ancho hacia la zona apical. El disco de fijación es pequeño, redondo de forma irregular.
Hábitat: se encuentra principalmente en el intermareal medio-alto en pozas con fondo arenoso.
Estado reproductivo: no se observa estructuras, por lo que se encuentra en una etapa vegetativa.

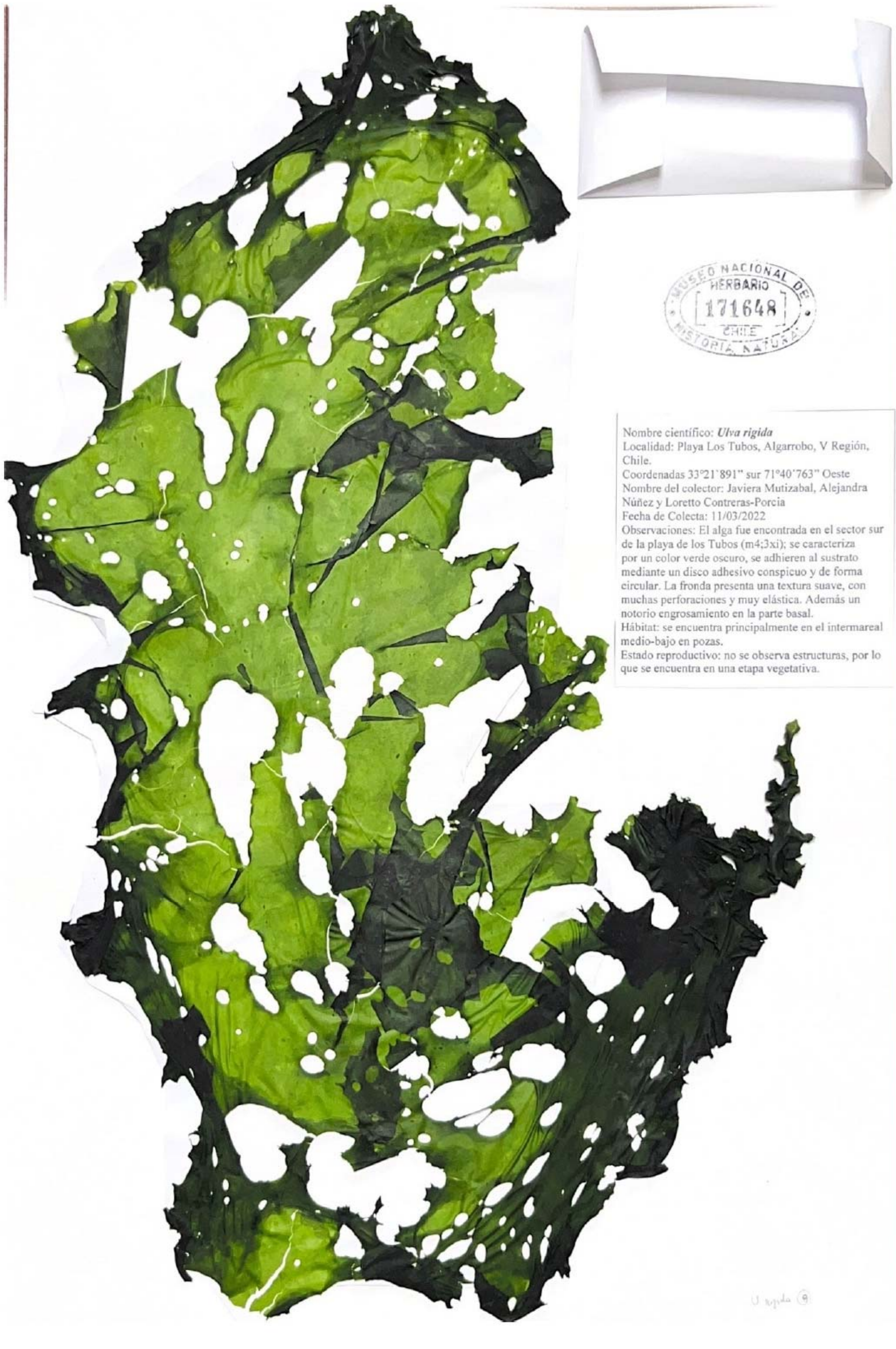

MUSEO NACIONAL DE HISTORIA NATURAL
HERBARIO
171648
CHILE

Nombre científico: *Ulva rigida*
Localidad: Playa Los Tubos, Algarrobo, V Región, Chile.
Coordenadas 33°21`891" sur 71°40`763" Oeste
Nombre del colector: Javiera Mutizabal, Alejandra Núñez y Loretto Contreras-Porcia
Fecha de Colecta: 11/03/2022
Observaciones: El alga fue encontrada en el sector sur de la playa de los Tubos (m4;3xi); se caracteriza por un color verde oscuro, se adhieren al sustrato mediante un disco adhesivo conspicuo y de forma circular. La fronda presenta una textura suave, con muchas perforaciones y muy elástica. Además un notorio engrosamiento en la parte basal.
Hábitat: se encuentra principalmente en el intermareal medio-bajo en pozas.
Estado reproductivo: no se observa estructuras, por lo que se encuentra en una etapa vegetativa.

U. rigida (9)

MUSEO NACIONAL DE HISTORIA NATURAL
HERBARIO
171650
CHILE

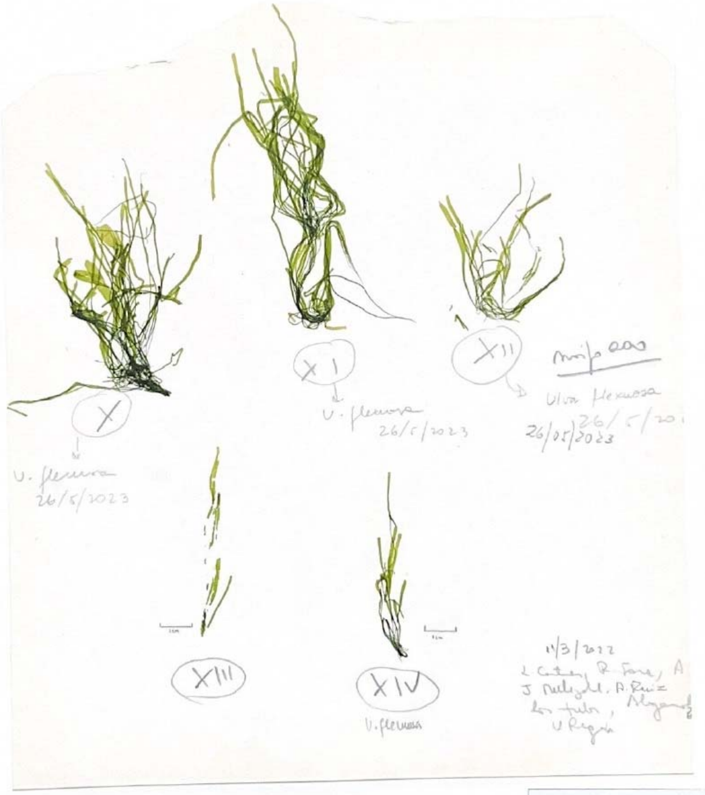

Nombre científico: ***Ulva flexuosa***
Localidad: Playa Los Tubos, Algarrobo, V Región, Chile.
Coordenadas 33°21´891" sur 71°40´763" Oeste
Nombre del colector: Javiera Mutizabal, Alejandra Núñez y Loretto Contreras-Porcia
Fecha de Colecta: 11/03/2022
Observaciones: El alga fue encontrada en el sector su de la playa de Los Tubos (X-XIV); se caracterizan por presentar un talo tubular de color verde claro, delgado, manteniendo su grosor desde el disco hasta la zona apical y en algunos casos ligeramente más ancho hacia la zona apical. El disco de fijación es pequeño, redondo de forma irregular.
Hábitat: se encuentra principalmente en el intermareal medio-alto en pozas con fondo arenoso.
Estado reproductivo: no se observa estructuras, por lo que se encuentra en una etapa vegetativa.

MUSEO NACIONAL DE HISTORIA NATURAL
HERBARIO
171646
CHILE

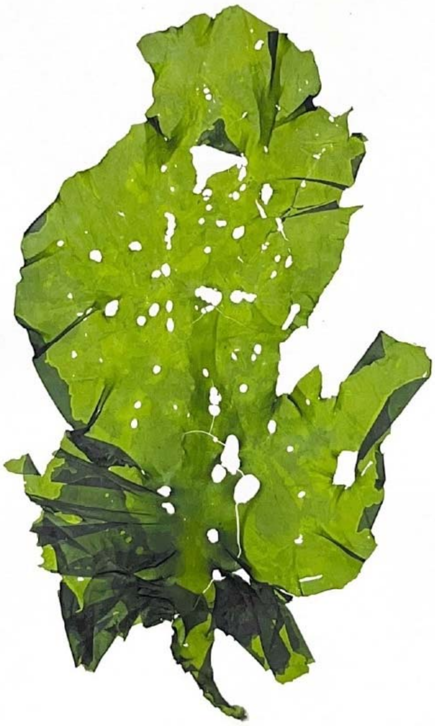

Nombre científico: ***Ulva rigida***
Localidad: Playa Los Tubos, Algarrobo, V Región, Chile.
Coordenadas 33°21´891" sur 71°40'763" Oeste
Nombre del colector: Javiera Mutizabal, Alejandra Núñez y Loretto Contreras-Porcia
Fecha de Colecta: 11/03/2022
Observaciones: El alga fue encontrada en el sector sur de la playa de los Tubos (m1-Fcm); se caracteriza por un color verde oscuro, se adhieren al sustrato mediante un disco adhesivo conspicuo y de forma circular. La fronda presenta una textura suave, con perforaciones y muy elástica. Además un notorio engrosamiento en la parte basal.
Hábitat: se encuentra principalmente en el intermareal medio-bajo en pozas.
Estado reproductivo: no se observa estructuras, por lo que se encuentra en una etapa vegetativa.

MUSEO NACIONAL DE HISTORIA NATURAL
HERBARIO
171645
CHILE

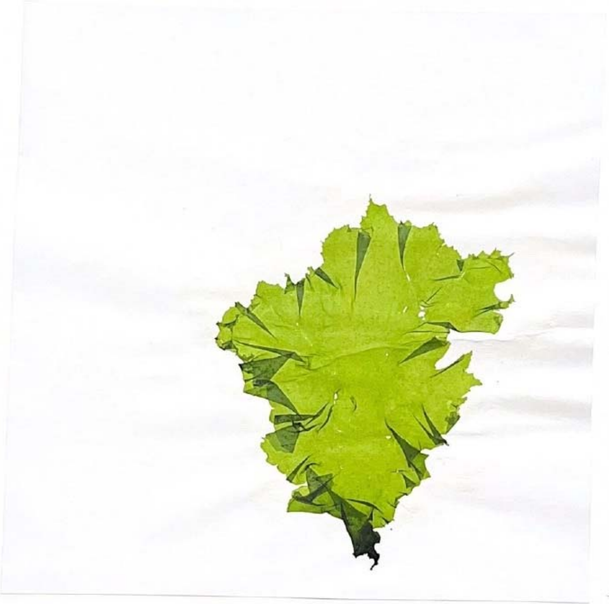

Nombre científico: *Ulva rigida*
Localidad: Playa Los Tubos, Algarrobo, V Región, Chile.
Coordenadas 33°21'891" sur 71°40'763" Oeste
Nombre del colector: Javiera Mutizabal, Alejandra Núñez y Loretto Contreras-Porcia
Fecha de Colecta: 11/03/2022
Observaciones: El alga fue encontrada en el sector sur de la playa de los Tubos (m2-XI); se caracteriza por un color verde oscuro, se adhieren al sustrato mediante un disco adhesivo conspicuo y de forma circular. La fronda presenta una textura suave, con perforaciones y muy elástica. Además un notorio engrosamiento en la parte basal.
Hábitat: se encuentra principalmente en el intermareal medio-bajo en pozas.
Estado reproductivo: no se observa estructuras, por lo que se encuentra en una etapa vegetativa.

MUSEO NACIONAL DE HISTORIA NATURAL
HERBARIO
171644
CHILE

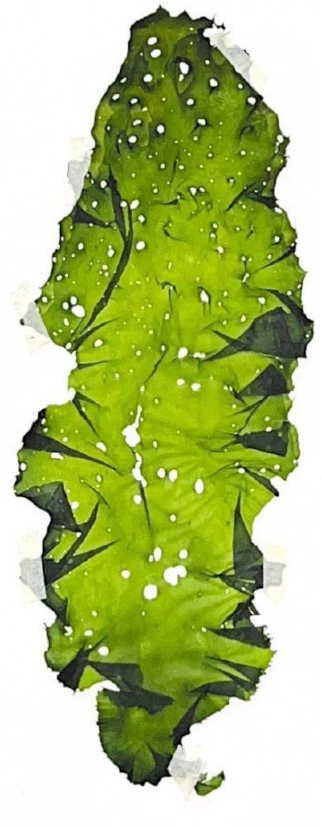

Nombre científico: *Ulva rigida*
Localidad : Playa Los Tubos, Algarrobo, V Región, Chile.
Coordenadas 33°21`891" sur 71°40`763" Oeste
Nombre del colector: Javiera Mutizabal, Alejandra Núñez y Loretto Contreras-Porcia
Fecha de Colecta: 11/03/2022
Observaciones: El alga fue encontrada en el sector sur de la playa de los Tubos (m3-XII); se caracteriza por un color verde oscuro, se adhieren al sustrato mediante un disco adhesivo conspicuo y de forma circular. La fronda presenta una textura suave, con perforaciones y muy elástica. Además un notorio engrosamiento en la parte basal.
Hábitat: se encuentra principalmente en el intermareal medio-bajo en pozas.
Estado reproductivo: no se observa estructuras, por lo que se encuentra en una etapa vegetativa.

MUSEO NACIONAL DE HISTORIA NATURAL
HERBARIO
171642
CHILE

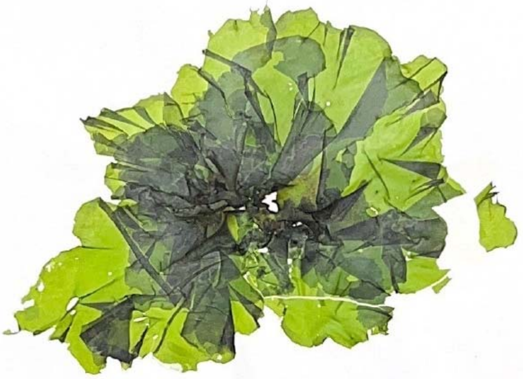

Nombre científico: ***Ulva rigida***
Localidad: Playa Los Tubos, Algarrobo, V Región, Chile.
Coordenadas 33°21`891" sur 71°40`763" Oeste
Nombre del colector: Javiera Mutizabal, Alejandra Núñez y Loretto Contreras-Porcia
Fecha de Colecta: 11/03/2022
Observaciones: El alga fue encontrada fuera del transecto Fc1 del sector sur de la playa de los Tubos; se caracteriza por un color verde oscuro, se adhieren al sustrato mediante un disco adhesivo conspicuo y de forma circular. La fronda presenta una textura suave, con perforaciones y muy elástica. Además un notorio engrosamiento en la parte basal.
Hábitat: se encuentra principalmente en el intermareal medio-bajo en pozas.
Estado reproductivo: no se observa estructuras, por lo que se encuentra en una etapa vegetativa.

MUSEO NACIONAL DE HISTORIA NATURAL
HERBARIO
171643
CHILE

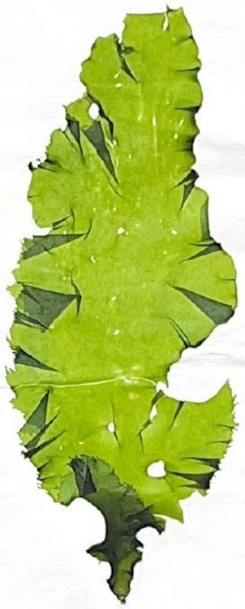

Nombre científico: *Ulva rigida*
Localidad: Playa Los Tubos, Algarrobo, V Región, Chile.
Coordenadas 33°21´891" sur 71°40´763" Oeste
Nombre del colector: Javiera Mutizabal, Alejandra Núñez y Loretto Contreras-Porcia
Fecha de Colecta: 11/03/2022
Observaciones: El alga fue encontrada en el sector sur de la playa de los Tubos (m2-X); se caracteriza por un color verde oscuro, se adhieren al sustrato mediante un disco adhesivo conspicuo y de forma circular. La fronda presenta una textura suave, con perforaciones y muy elástica. Además un notorio engrosamiento en la parte basal.
Hábitat: se encuentra principalmente en el intermareal medio alto en pozas con sustrato rocoso.
Estado reproductivo: no se observa estructuras, por lo que se encuentra en una etapa vegetativa.

MUSEO NACIONAL DE HISTORIA NATURAL
HERBARIO
171647
CHILE

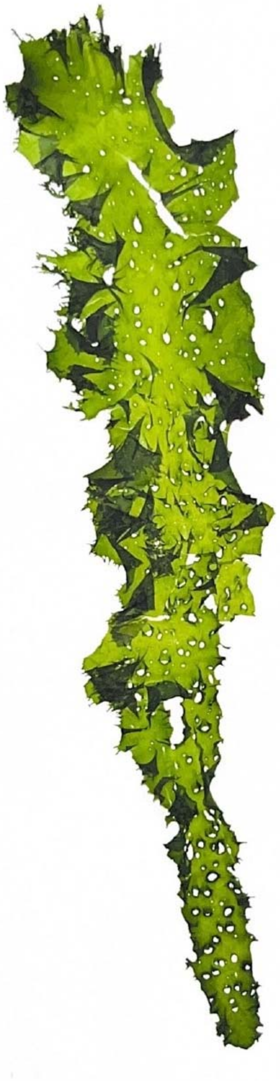

Nombre científico: ***Ulva rigida***
Localidad: Playa Los Tubos, Algarrobo, V Región, Chile.
Coordenadas 33°21'891" sur 71°40'763" Oeste
Nombre del colector: Javiera Mutizabal, Alejandra Núñez y Loretto Contreras-Porcia
Fecha de Colecta: 11/03/2022
Observaciones: El alga fue encontrada en el sector sur de la playa de los Tubos (m4;3x); se caracteriza por un color verde oscuro, se adhieren al sustrato mediante un disco adhesivo conspicuo y de forma circular. La fronda presenta una textura suave, con pequeñas perforaciones y muy elástica. Además un notorio engrosamiento en la parte basal. También se puede observar pequeñas
Hábitat: se encuentra principalmente en el intermareal medio-bajo en pozas.
Estado reproductivo: no se observa estructuras, por lo que se encuentra en una etapa vegetativa.

MUSEO NACIONAL DE HISTORIA NATURAL
HERBARIO
171641
CHILE

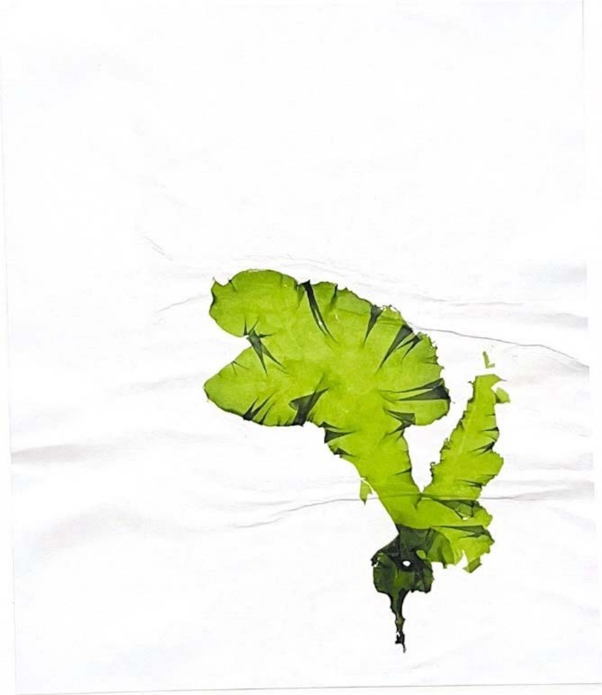

Nombre científico: ***Ulva rigida***
Localidad: Playa Los Tubos, Algarrobo, V Región Chile.
Coordenadas 33°21`891" sur 71°40´763" Oeste
Nombre del colector: Javiera Mutizabal, Loretto Contreras-Porcia y Alejandra Núñez.
Fecha de Colecta: 23/08/2021
Observaciones: El alga fue encontrada en el sector sur de la playa de los Tubos (m1-x); se caracteriza por un color verde oscuro, se adhieren al sustrato mediante un disco adhesivo conspicuo y de forma circular. La fronda presenta una textura suave, con perforaciones y muy elástica. Además un notorio engrosamiento en la parte basal.
Hábitat: se encuentra principalmente en el intermareal medio-bajo en pozas.
Estado reproductivo: no se observa estructuras, por lo que se encuentra en una etapa vegetativa.

MUSEO NACIONAL DE HISTORIA NATURAL
HERBARIO
171640
CHILE

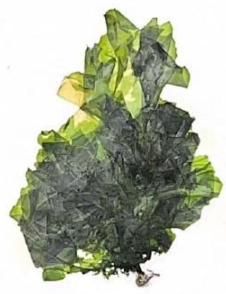

Nombre científico: ***Ulva rigida***
Localidad: Playa Los Tubos, Algarrobo, V Región, Chile.
Coordenadas 33°21`891" sur 71°40´763" Oeste
Nombre del colector: Javiera Mutizabal, Florentina Piña y Loretto Contreras-Porcia
Fecha de Colecta: 11/03/2022
Observaciones: El alga fue encontrada en el cuadrante 52 transecto 1, sector sur de la playa de los Tubos; se caracteriza por un color verde oscuro, se adhieren al sustrato mediante un disco adhesivo conspicuo y de forma circular. La fronda presenta una textura suave, con perforaciones y muy elástica. Además un notorio engrosamiento en la parte basal.
Hábitat: se encuentra principalmente en el intermareal medio-bajo en pozas.
Estado reproductivo: no se observa estructuras, por lo que se encuentra en una etapa vegetativa.

MUSEO NACIONAL DE HERBARIO
171639
CHILE
HISTORIA NATURAL

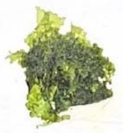

Nombre científico: ***Ulva stenophylloides***
Localidad: Playa Los Tubos, Algarrobo, V Región, Chile.
Coordenadas 33°21'891" sur 71°40'763" Oeste
Nombre del colector: Javiera Mutizabal, Alejandra Núñez y Loretto Contreras-Porcia
Fecha de Colecta: 11/03/2022
Observaciones: El alga fue encontrada en el cuadrante 25 en el transecto 1, en el sector sur de la playa de los tubos; se caracterizaba por una fronda suave al tacto de color verde claro, sus frondas son de forma variable, no presenta un estipe definido.
Hábitat: se encuentra principalmente en el intermareal medio bajo.
Estado reproductivo: no se observa estructuras, por lo que se encuentra en una etapa vegetativa.

MUSEO NACIONAL DE HISTORIA NATURAL
HERBARIO
171638
CHILE

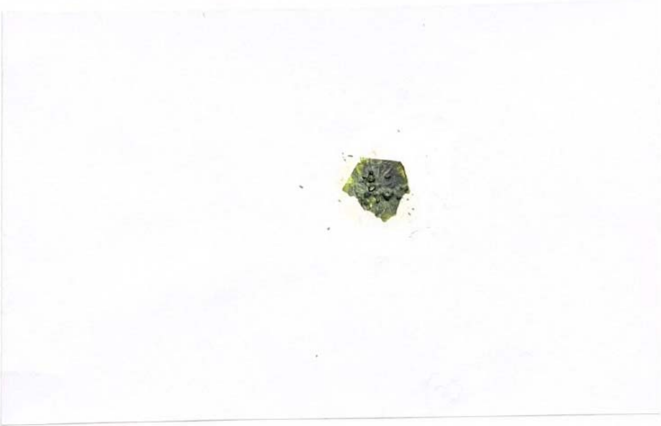

Nombre científico: ***Ulva stenophylloides***
Localidad: Playa Los Tubos, Algarrobo, V Región, Chile.
Coordenadas 33º21'891" sur 71º40'763" Oeste
Nombre del colector: Javiera Mutizabal, Alejandra Núñez y Loretto Contreras-Porcia
Fecha de Colecta: 11/03/2022
Observaciones: El alga fue encontrada en el cuadrante 23 en la transecta 1 en el sector sur de la playa de los tubos; se caracterizaba por una fronda suave al tacto de color verde claro, sus frondas son de forma variable, no presenta un estipe definido.
Hábitat: se encuentra principalmente en el intermareal medio en pozas.
Estado reproductivo: no se observa estructuras, por lo que se encuentra en una etapa vegetativa.

MUSEO NACIONAL DE HISTORIA NATURAL
HERBARIO
171637
CHILE

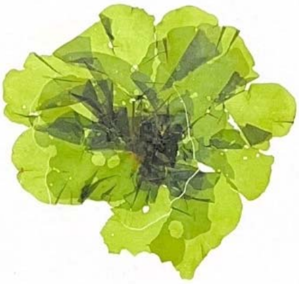

Nombre científico: ***Ulva stenophylloides***
Localidad: Playa Los Tubos, Algarrobo, Chile.
Coordenadas 33°21`891" sur 71°40`763" Oeste
Nombre del colector: Florentina Piña, Javiera Mutizabal, Loretto Contreras-Porcia
Fecha de Colecta: 23/08/2021
Observaciones: El alga fue encontrada en el transecto 1 cuadrante 9 en el sector sur de la playa de los tubos; se caracterizaba por una fronda suave al tacto de color verde claro, sus frondas son de forma variable, no presenta un estipe definido
Hábitat: se encuentra principalmente en el intermareal alto y medio en pozas.
Estado reproductivo: no se observa estructuras, por lo que se encuentra en una etapa vegetativa.

MUSEO NACIONAL DE HISTORIA NATURAL
HERBARIO
171636
CHILE

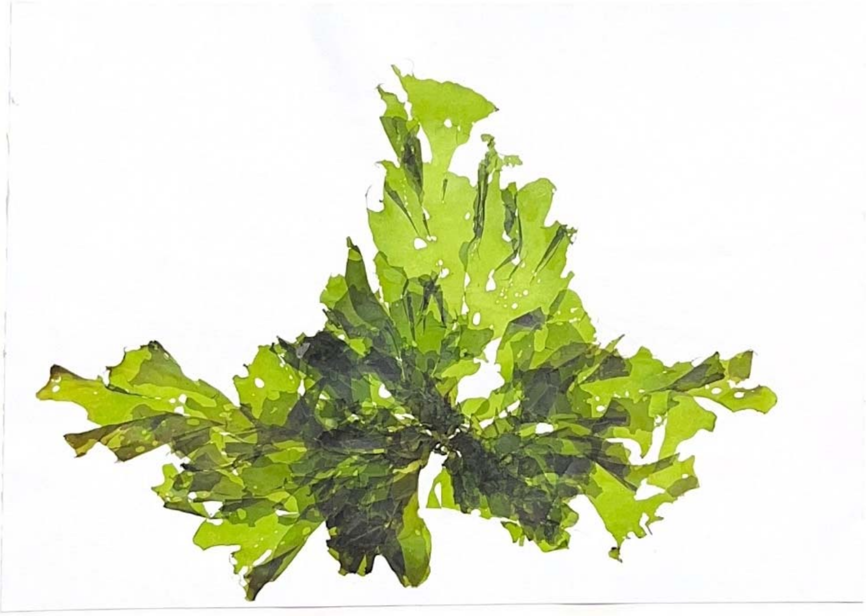

Nombre científico: ***Ulva stenophylloides***
Localidad: Playa Los Tubos, Algarrobo, Chile.
Coordenadas 33°21´891" sur 71°40´763" Oeste
Nombre del colector: Florentina Piña, Javiera Mutizabal, Loretto Contreras-Porcia
Fecha de Colecta: 23/08/2021
Observaciones: El alga fue encontrada en el transecto 1 cuadrante 1 en el sector sur de la playa los Tubos. Presenta una lámina de forma foliosa con pequeñas perforaciones.
Hábitat: Intermareal medio, se fijan principalmente enzonas donde hay más presencia de arena y no de rocas-
Estado reproductivo no se observó estructuras reproductivas, por lo que podría estar en una fase vegetativa.
