# Supplementary material for: Morphological and Molecular Identification of Ulva spp. (Ulvophyceae; Chlorophyta) from Algarrobo Bay, Chile: Understanding the Composition of Green Tides
Source: Plants (Basel). 2024 Apr 30;13(9):1258. doi: 10.3390/plants13091258 (PMC11085182; doi:10.3390/plants13091258)
Supplement: Supplementary file 1 [file plants-13-01258-s001.zip › Table S1.pdf]

Table S1. Isolate code, specimen_voucher numbers and GENBANK accession numbers of *Ulva stenophylloides*, *Ulva uncialis*, *Ulva compressa* and *Ulva aragoensis* from Algarrobo beach, Valparaíso, Chile, sequenced (*ITS1*, and *tuf*A markers) during this work.

Isolate code	Species	Specimen_voucher number	GENBANK accession number	
			ITS	*tuf*A
Sample1	*Ulva stenophylloides*	SGO171638	OR514517	-
Sample2	*Ulva stenophylloides*	-	OR514518	-
Sample5	*Ulva uncialis*	-	OR514519	-
Sample7	*Ulva uncialis*	-	OR514520	-
Sample8	*Ulva uncialis*	-	OR514521	-
Sample9	*Ulva uncialis*	SGO171642	OR514522	-
Sample10	*Ulva uncialis*	-	OR514523	.
Sample2E	*Ulva stenophylloides*	SGO171636	OR514524	OR526355
Sample2I	*Ulva stenophylloides*	SGO171637	OR514525	-
Sample2J	*Ulva stenophylloides*	-	OR514526	OR526358
Sample2XI	*Ulva uncialis*	SGO171645	OR514527	-
Sample2XII	*Ulva uncialis*	-	OR514528	-
Sample3D	*Ulva stenophylloides*	-	OR514529	-
Sample3E	*Ulva stenophylloides*	SGO171639	OR514530	-
Sample3G	*Ulva stenophylloides*	-	OR514531	-
Sample3XI	*Ulva uncialis*	SGO171648	OR514532	-
Sample3XII	*Ulva uncialis*	SGO171644	OR514533	-
Sample4A	*Ulva uncialis*	-	OR514534	-
Sample4C	*Ulva uncialis*	-	OR514535	-
Sample4D	*Ulva uncialis*	-	OR514536	OR526362
Sample4E	*Ulva uncialis*	-	OR514537	-
Sample4G	*Ulva uncialis*	-	OR514538	OR526364

Sample4M	*Ulva uncialis*	-	OR514539	-
Sample4X	*Ulva uncialis*	-	OR514540	-
Sample4XII	*Ulva uncialis*	-	OR514541	-
Sample8AM	*Ulva uncialis*	-	OR514542	OR526367
Sample100X	*Ulva compressa*	-	OR514543	-
Sample100XII	*Ulva compressa*	-	OR514544	-
Sample100XIII	*Ulva compressa*	SGO171649	OR514545	-
Sample100XIV	*Ulva compressa*	-	OR514546	-
Sample200X	*Ulva aragoensis*	-	OR514547	-
Sample200XI	*Ulva aragoensis*	-	OR514548	-
Sample200XII	*Ulva aragoensis*	SGO171650	OR514549	-
Sample300XI	*Ulva aragoensis*	-	OR514550	-
Sample300XII	*Ulva aragoensis*	SGO171651	OR514551	-
Sample300XIV	*Ulva compressa*	-	OR514552	-
Sample4AM	*Ulva australis*	SGO171635	OR514553	OR526361
SampleFCMM	*Ulva uncialis*	-	OR514554	-
Sample6	*Ulva uncialis*	SGO171640	OR514555	OR526366
Sample1X	*Ulva uncialis*	SGO171641	OR514556	-
Sample2X	*Ulva uncialis*	SGO171643	OR514557	-
Sample3X	*Ulva uncialis*	SGO171647	OR514558	-
Sample2G	*Ulva stenophylloides*	-	-	OR526356
Sample2H	*Ulva stenophylloides*	-	-	OR526357
Sample3H	*Ulva stenophylloides*	-	-	OR526359
Sample3K	*Ulva stenophylloides*	-	-	OR526360
Sample4F	*Ulva uncialis*	-	-	OR526363
Sample4H	*Ulva uncialis*	-	-	OR526365
